# Integrated workflow for discovery of microprotein-coding small open reading frames

**DOI:** 10.1016/j.xpro.2023.102649

**Published:** 2023-10-23

**Authors:** Kevin Cao, Yasamin Hajy Heydary, Gregory Tong, Thomas Farid Martinez

**Affiliations:** 1 Department of Pharmaceutical Sciences, University of California, Irvine, Irvine, CA 92617, USA; 2 Department of Biological Chemistry, University of California, Irvine, Irvine, CA 92617, USA

**Keywords:** Bioinformatics, Sequence Analysis, Cell Biology, Cell Culture, Genomics, Sequencing, RNAseq, Molecular Biology, Gene Expression

## Abstract

Small open reading frame (smORF)-encoded microproteins, proteins containing less than 100–150 amino acids, are an emerging class of functional biomolecules. Here, we present a protocol for identifying translated smORFs in mammalian systems genome wide. We describe steps for generation of ribosome profiling (Ribo-seq) data, *in silico* translation of a transcriptome assembly to create an ORF database, and computational analysis of Ribo-seq to score individual smORFs for translation. Identification of translated smORFs is the first step to studying the functions of microproteins.

For complete details on the use and execution of this protocol, please refer to Martinez et al.^1^

## Highlights

• Preparation of high-quality Ribo-seq libraries
• Processing, aligning, and quality checks for sequenced Ribo-seq data
• Creation of ORF database from a transcriptome to use in translation prediction
• Scoring of small ORF translation using RibORF software package

## Abstract (editor highlights)

Publisher’s note: Undertaking any experimental protocol requires adherence to local institutional guidelines for laboratory safety and ethics.

## Graphical abstract

**Figure:**
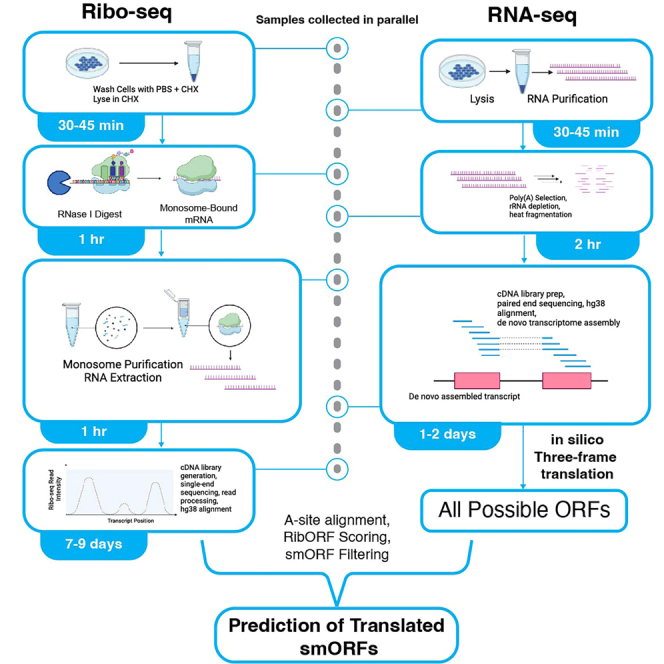

## Before you begin

This protocol outlines steps for differentiated mouse adipocytes, which are adherent, but the steps described here can be applied to other mammalian cell types including those grown in suspension. Before beginning the protocol, ensure that all materials listed in the “key resources table” and “materials and equipment” sections are ready to use.

Executing this protocol in an RNase-free environment is critical for successful completion of sample extraction and library preparation. Make sure that all materials used are certified as RNase-free. We also suggest using an RNase decontamination solution such as RNaseZap to clean work spaces and tools before starting. Additionally, maintaining RNA stability is crucial. Therefore, purified RNA intermediates are recommended to be processed immediately and handled on ice. If long-term storage of RNA extracted for this protocol is required, they should be kept at −80°C, otherwise short-term storage for one to two days at −20°C is acceptable.

### Cell culture and expansion

**Timing: <1 week**

Cells are grown up to scale to yield sufficient polysomes for Ribo-seq.

1. Culture adherent cells to 80%–90% confluency in a 10-cm or 15-cm diameter cell culture dish for harvesting ribosome footprints on day 1.

**CRITICAL:** An adequate number of cells must be grown to ensure the recovery of a sufficient amount of input polysomes (troubleshooting 1).

***Note:*** For most cell types we have tested, beginning with 40–60 μg of total RNA in lysate will yield the 1–5 μg of isolated ribosome-protected fragment (RPF) RNA needed for library completion.

### Buffer preparation

**Timing: 30 min**

Buffers are prepared fresh for each Ribo-seq experiment.

2. Prepare buffers fresh on the day it is required for the protocol. Stock buffers are listed below and should be prepared with RNase/DNase-free molecular-grade water and reagents.

## Key resources table

**Table:** 

REAGENT or RESOURCE	SOURCE	IDENTIFIER
**Chemicals, peptides, and recombinant proteins**		

Cycloheximide (CHX)	Thermo Fisher	Cat# J66004XF
Phosphate buffered saline (PBS)	Genesee Scientific	Cat# 25-507
Triton X-100 stock solution (20% v/v)	Cayman Chemical Company	Cat# 600217
SDS solution, molecular biology grade (10% w/v)	Promega	Cat# V6551
Teknova 1 M TRIS-HCL PH 7.4 1 L	Fisher Scientific	Cat # 50-843-265
Sodium chloride	Fisher Scientific	Cat # 722716
Magnesium chloride	G-Biosciences	Cat# R004
Dithiothreitol (DTT)	Genesee Scientific	Cat # 18-203
cOmplete™ ULTRA Tablets, Mini, EDTA-free, EASYpack Protease Inhibitor Cocktail	Roche	Cat# 05892791001
Corning Incorporated Molecular Biology Grade Water Tested to USP Sterile water, Corning™	Corning Incorporated	Cat# MT46000CM
Polyethylene glycol solution 40% (w/w)	Millipore Sigma	Cat# P1458-25ML
Promega RNasin™ Plus RNase Inhibitor	Promega	Cat# PRN2615
SUPERase⋅In™ RNase Inhibitor (20 U/μL)	Invitrogen	Cat# AM2696
Acid-phenol:chloroform, pH 4.5 (with isoamyl alcohol, 125:24:1)	Invitrogen	Cat# AM9720
Glycogen, molecular biology grade	Thermo Fisher	Cat# R0561
Isopropanol, 99.5%, for molecular biology, DNAse, RNAse, and protease free	Thermo Fisher	Cat# 327270010
SYBR Gold nucleic acid gel stain	Invitrogen	Cat# S11494
Ethyl alcohol, pure	Sigma-Aldrich	Cat# E7023
Sodium acetate (3 M), pH 5.5, RNase-free	Invitrogen	Cat# AM9740
TBE 10× Liquid Solution, ultra-pure grade solution	Genesee Scientific	Cat # 20-196
Novex™ TBE-Urea Sample Buffer (2×)	Invitrogen	Cat# LC6876
Novex™ Hi-Density TBE Sample Buffer (5×)	Invitrogen	Cat# LC6678
GlycoBlue™ Coprecipitant	Invitrogen	Cat# AM9516

**Critical commercial assays**		

Human/Mouse/Rat riboPOOL for ribosome profiling samples 24 rxn	siTOOLs Biotech	Cat# dp-K024-000050
Zymo Research R1016 RNA Clean & Concentrator-5, Zymo Research Kit, 200 Preps/Unit	Zymo Research	Cat# 11-326
Qubit™ RNA Broad Range (BR) Assay Kit	Invitrogen	Cat# Q10211
Qubit™ dsDNA HS Assay Kit	Invitrogen	Cat# Q32851
5′ DNA adenylation kit	New England Biolabs	Cat# E2610S
Phusion Hot Start II DNA Polymerase	Thermo Fisher	Cat# F549S

**Oligonucleotides**		

**Note:** All custom oligos are HPLC purified		
27 & 30 nt RNA markers	Integrated DNA Technologies	Calviello et al.^2^
20/100 oligo ladder	Integrated DNA Technologies	51-05-15-02
Thermo Scientific™ dNTP Mix (10 mM each)	Fisher Scientific	Cat# FERR0192
Ribo 3′ adapter 5’-/P/-AGATCGGAAGAGCACACGTCTGAA-/3ddC/-3′	Integrated DNA Technologies	N/A
Ribo RT Primer 5’-/Phos/-AGATCGGAAGAGCGTCGTGT AGGGAAAGAG-/iSp18/-GTGACTGGAG TTCAGACGTGTGCTC-3′	Integrated DNA Technologies	N/A
Ribo PCR Forward Primer 5′-AATGATACGGCGACCACCGAGATCT ACACTCTTTCCCTACACGACGCTC-3′	Integrated DNA Technologies	N/A
Ribo PCR Reverse Index Primer (N denotes barcode sequence) 5′-CAAGCAGAAGACGGCATACGAGAT**NNNNNN**G TGACTGGAGTTCAGACGTGTGCTCTTCCGATCT-3′	Integrated DNA Technologies	N/A

**Other**		

RNaseZap™ RNase Decontamination Solution	Invitrogen	Cat# AM9780
Thermo Scientific™ Sorvall™ Legend™ Micro 17R Microcentrifuge	Thermo Fisher	Cat# 75 772 441
Qubit™ 4 Fluorometer, with WiFi	Invitrogen	Cat# Q33238
Benchmark Scientific R5010 Rotating Mixer, w/ Rotisseries for 1.5/2.0, 15	Genesee Scientific	Cat# 31-439
NanoDrop™ 2000c Spectrophotometer	Thermo Fisher	Cat# ND-2000C
VORTEX-GENIE® 2	USA Scientific	Cat# 7404-5600
Seal-Rite 0.5 mL graduated microcentrifuge tube, natural	USA Scientific	Cat# 1605-0000
Seal-Rite 1.5 mL graduated microcentrifuge tube, natural	USA Scientific	Cat# 1615-5500
Ultrafree®-MC GV Centrifugal Filter	Millipore	Cat# UFC30GV0S
Cytiva MicroSpin™ S-400 HR Columns	Cytivia	Cat# GE27-5140-01
HSW® Norm-Ject® Sterile Luer-Slip Syringes, 1 mL	Air-Tite	Cat# 4010.200V0
NEEDLE 18G X 1IN PK100	VWR	Cat# 89219-294
NEEDLE 26G X 1/2IN PK100	VWR	Cat# 89219-324
VWR® Razor Blades	VWR	Cat# 55411-050
XCell SureLock™ Mini-Cell	Invitrogen	Cat# EI0001
PowerEase™ Touch 120W Power Supply (115 VAC)	Invitrogen	Cat# PS0120
Apex 100bp-Low DNA Ladder, 100bp-1kb, 1 mL/Unit	Genesee Scientific	Cat# 19-109
Novex™ TBE-Urea Gels, 15%, 10 well	Invitrogen	Cat# EC6885BOX
Novex™ TBE-Urea Gels, 10%, 10 well	Invitrogen	Cat# EC6875BOX
Novex™ TBE Gels, 8%, 10 well	Invitrogen	Cat# EC6215BOX
Biometra TRIO 48 Endpoint Thermal Cycler	Analytik Jena	Cat# 846-2-070-720
Eppendorf ThermoMixer® C	Eppendorf	Cat# 5382000023
MagJET Separation Rack	Thermo Fisher	Cat# MR02
Agencourt® AMPure® XP - 5 mL	Beckman Coulter	Cat# A63880
Dual LED Blue/White Light Transilluminator	Invitrogen	Cat# LB0100
Invitrogen TURBO Dnase (2U/uL)	Invitrogen	Cat# AM2238
*E. coli* RNase I	Lucigen	Cat# N6901K
EpiScript RNase H- Reverse Transcriptase Kit	Lucigen	Cat# ERT12910K
CircLigase™ ssDNA Ligase	Lucigen	Cat# CL4111K
T4 polynucleotide kinase (PNK)	Lucigen	Cat# P0503K
5′ Deadenylase	New England Biolabs	Cat# M0331S
RecJ Exonuclease	Lucigen	Cat# RJ411250
Exonuclease I, *E. coli*	Lucigen	Cat# X40520K
Hybridase™ Thermostable RNase H	Lucigen	Cat# H39500
T4 RNA Ligase 2	Lucigen	Cat# LR2D1132K
CO₂ incubators with hot air sterilization, CB-S series, Binder	Binder	Cat# CB-S-170
Olympus Plastics 24–154, 0.2 mL individual PCR tubes flat cap, natural, bag of 1000 tubes/unit	Olympus	Cat# 24-154

**Software and algorithms**		

STAR v2.7.9a	Dobin et al.^3^	https://github.com/alexdobin/STAR
RibORF v0.1	Ji et al.^4^	https://github.com/zhejilab/RibORF
HOMER	Heinz et al.^5^	http://homer.ucsd.edu/homer/
FASTX toolkit v0.0.14	hannonlab.cshl.edu	http://hannonlab.cshl.edu/fastx_toolkit/
Samtools v1.15.1	Li et al.^6^	http://www.htslib.org/
Python v2.7.18	https://www.anaconda.com/products/individual	https://www.anaconda.com/products/individual
Bedtools v2.30.0	Quinlan and Hall^7^	https://github.com/arq5x/bedtools2
ncbi-blast	https://ftp.ncbi.nlm.nih.gov/blast/executables/blast+/LATEST/	https://ftp.ncbi.nlm.nih.gov/blast/executables/blast+/LATEST/
R v4.1.2	https://www.r-project.org/	https://www.r-project.org/
GTFtoFASTA	Martinez et al.^8^	N/A
UCSC tools	UCSC Genome Browser	http://hgdownload.cse.ucsc.edu/admin/exe/linux.x86_64/

## Materials and equipment

**Table:** 1× Lysis Buffer

Reagent	Final concentration	Amount
20% Triton X-100	1%	500 μL
1 M Tris pH 7.4	20 mM	200 μL
1 M NaCl_2_	150 mM	1.5 mL
1 M MgCl_2_	5 mM	50 μL
Nuclease-free water	N/A	7.605 mL
CHX	100 μg/mL	10 μL
1 M DTT	1 mM	10 μL
Turbo DNase	25 U/mL	125 μL
Roche ULTRA Protease Inhibitor Cocktail	1×	1 tablet
**Total**	**N/A**	**10 mL**

**Table:** S-400 Column Wash Buffer

Reagent	Final concentration	Amount
1 M Tris pH 7.4	20 mM	180 μL
1 M NaCl_2_	150 mM	1.35 mL
1 M MgCl_2_	5 mM	45 μL
Promega RNasin Plus Inhibitor	20 U/mL	2.25 μL
Nuclease-free water	N/A	7.423 mL
**Total**	**N/A**	**9 mL**

## Step-by-step method details

### Cycloheximide treatment & lysate preparation

**Timing: 45 min**

Cells are treated with cycloheximide in the wash and lysis buffers to inhibit translation elongation and stall ribosomes.^9^

1. Wash cells with 5 mL of ice-cold PBS containing 100 μg/mL Cycloheximide (CHX) for a 10 cm plate and **immediately** remove as much PBS as possible.

***Note:*** The longer incubations with CHX in the PBS wash can cause accumulation of ribosomes near the start site of ORFs, representing an artifact of the CHX treatment rather than native translation conditions. For suspension cell cultures, we recommend minimizing the incubation time with CHX by spinning down the cells in PBS with CHX for as short a time as possible, e.g., 3 min.

2. Add 400 μL of ice-cold lysis buffer to the plate and scrape cells off in the lysis buffer and collect the buffer and cells into a fresh tube.

***Note:*** Prepare lysis buffer on the day of harvesting samples. The lysis buffer volume can be adjusted in order to achieve the optimal concentration of lysate RNA for digests and will depend on the cell type and number of cells (troubleshooting 1).

3. Vortex and/or pass your sample through a 26-gauge needle to homogenize the cells and let it incubate on ice for 10 min. Use of a probe sonicator is not recommended.
4. Clarify the lysate by spinning it at 15,000 × *g* for 10 min at 4°C.
5. Transfer supernatant to a new RNase-free tube.

**CRITICAL:** RNA in cellular lysate is vulnerable to degradation and ribosome dissociation. Handle samples on ice and proceed with digestion the day of lysis if possible. Samples that need to be stored for later, they should be flash frozen in liquid nitrogen and stored at −80°C for up to one week.

### MicroSpin S-400 HR column preparation

**Timing: 1 h**

Prior to column purification of monosomes, the column resin is equilibrated with wash buffer. This step should be started just before the digest with RNase I.

6. Prepare one S-400 HR column per 100 μL of lysate. For 60 μg of input RNA in 300 μL lysis buffer for RNase I digestion, three columns will be used for monosome isolation.
7. Remove the cap and seal from each column and begin washing with 500 μL S-400 column wash buffer.
  a. Discard the flow-through after each wash.
  b. Perform six washes per column for 3 mL total wash buffer per column.

***Note:*** Prepare column wash buffer on the day of digest. The wash buffer passes through the column very slowly with gravity. To facilitate faster flow-through of the buffer, push air through the top of the columns by pressing a gloved finger over the top of the column. Ensure that there are no bubbles in the column resin.

**CRITICAL:** On the last wash, allow enough buffer to pass through the column such that there is about 15% volume of buffer sitting on top of the resin bed.

8. Spin the column at 600 × *g* for 4 min.

**CRITICAL:** Do not let the resin bed in the columns dry out during the wash steps. Doing so will result in low yields of RPFs. Ideally the column washes will be complete just prior to RNase I digest step.

### RNase I digest

**Timing: 1 h and 15 min**

Polysomes are subjected to RNase I digestion, which degrades unprotected RNA to yield monosomes containing RNA footprints that only span the width of the single ribosome.

9. Dilute 1 μL of supernatant with 9 μL of molecular grade H_2_O to measure RNA concentration.
10. Use Qubit 2.0 Broad Range RNA assay to measure RNA concentration.
11. Digest 40–60 μg of RNA in 200–300 μL of lysate with 0.375 U/μg of RNase I for 50 min at 20°C–25°C while rotating.

***Note:*** Optimal RNase I concentration needs to be determined for each cell or tissue type being studied (troubleshooting 2).

12. After completing the digest, add 10 μL of (200 U) of SuperaseIn Rnase Inhibitor and keep the samples on ice.

### Monosome isolation and acid Phenol:Chloroform extraction

**Timing: 2 h and 30 min**

Digested monosomes are separated from free digested RNA and unprotected RNA using a size exclusion spin column.

Monosomes are isolated from free-digested RNA fragments in the cell lysate using MicroSpin S-400 HR columns. The small RNA RPFs are then extracted using acid phenol:chloroform (troubleshooting 3).

13. Add 100 μL of digest in a dropwise manner directly to the resin of each spin column. Do not pipette the digest onto the walls of the column.
14. Centrifuge the columns at 600 × *g* for 2 min.
15. Collect ∼125 μL of monosomes from each column and combine the eluates from all columns per sample.

***Note:*** The extra ∼25 μL volume collected is from the remaining wash buffer in the column.

**Figure 1:**
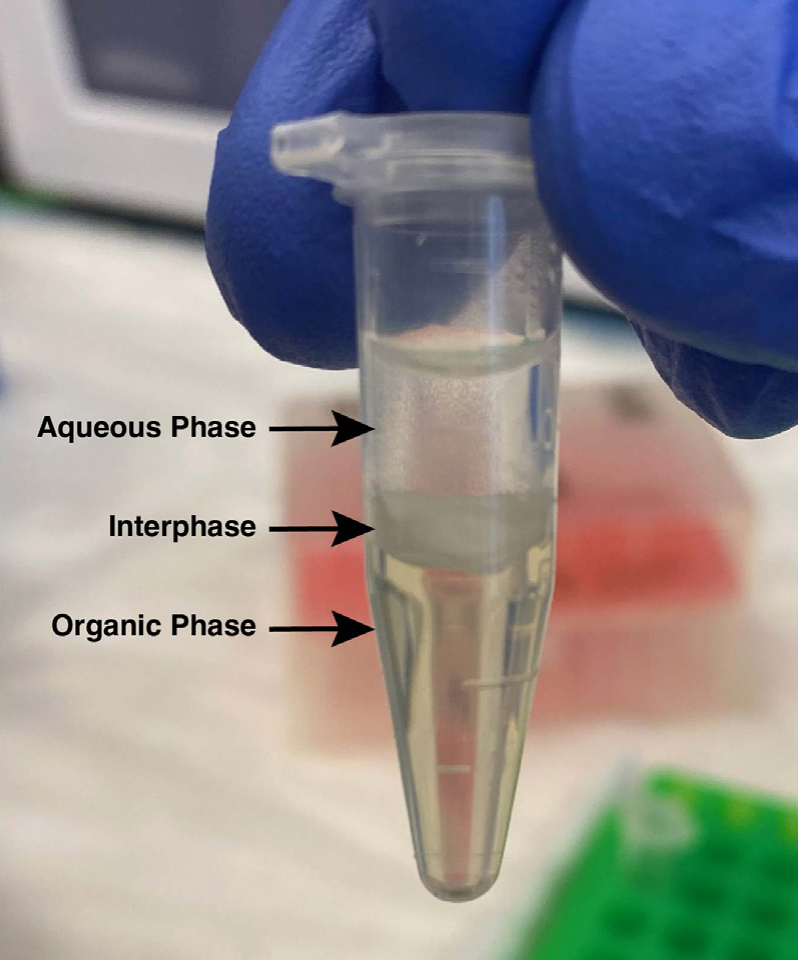
Acid phenol:chloroform extraction of RPFs Acid phenol:chloroform extraction separates RPFs and other RNA molecules from proteins, lipids, and DNA. Retrieve RPFs from the aqueous phase that settles in the top layer.
18. To each sample, add 1:10 volume of 3 M NaOAc, pH 5.2 and 2 μL of glycogen (15 mg/mL). Next, add 1 volume of ice-cold isopropanol. Crash out RPF RNA at −20°C for >2 h.

***Note:*** Glycogen is used as a carrier to trap nucleic acids during its precipitation, creating a more visible pellet.

16. Add an equal volume of acid phenol: chloroform to each sample, vortex the samples, and then incubate for 5 min at 20°C–25°C.
17. Spin the samples at >15,000 × *g* for 10 min at 4°C. Collect the aqueous phase from each sample after centrifuging while being careful not to pipette the interphase or organic phase (Figure 1).

19. Spin down the RPF RNA samples at >15,000 × *g* for 15 min at 4°C to pellet the RNA. Decant the supernatant, being careful not to disturb the pellet.
20. Wash the pellet once with ice-cold 80% ethanol. Centrifuge the samples again at >15,000 × *g* for 10 min at 4°C.
21. Decant the supernatant and allow the pellet to air dry for about 5–10 min. Do not over-dry the pellet while ensuring that the ethanol completely evaporates.
22. Resuspend the pellet in 15 μL of nuclease-free H_2_O. Use 1 μL of resuspended RNA to measure RNA concentration with a Nanodrop UV-Vis Spectrophotometer.

**Pause point:** Purified RNA can be stored for up to a week at −20°C or long-term at −80°C.

### rRNA depletion with siTOOLs Biotech riboPOOLs kit

**Timing: 1 h**

Contaminating ribosomal RNA (rRNAs) are removed from the sample in order to improve the depth of RPF sequencing. While there are many rRNA depletion kits commercially available, we recommend the siTOOLs riboPOOLs rRNA depletion kit for ribosome profiling.

#### rRNA probe hybridization

23. Adjust the sample to a final volume of 14 μL containing 1–5 μg of RNA with water.
24. Add 1 μL of rRNA probe to the sample.

***Note:*** siTOOLs riboPOOLs rRNA depletion kits have been observed to contain a varying concentration of rRNA probes between lots. It is recommended to measure the concentration of the probe prior to usage. We have found that 1.2×–1.5× concentrations of probe to RPF RNA is optimal for depleting rRNA (troubleshooting 4). Note that despite executing this step the majority of sequenced reads are likley to still be derived from rRNA sequences.

25. Add 5 μL of hybridization buffer (HB) and 1 μL of Promega RNasin Plus. Pipet up and down to mix well.
26. Incubate sample(s) for 10 min at 68°C in a thermocycler to denature RNA and allow samples to cool at a rate of 3 °C/min to 23°C.

#### Bead preparation

27. While your sample(s) are cooling down, resuspend the streptavidin-coated magnetic beads by vortexing them at medium speed.

***Note:*** For multiple samples, magnetic beads may be prepared by batch processing as in the manufacturer’s protocol.

28. Transfer 90 μL of the bead suspension per sample into a fresh microcentrifuge tube and place the tube on a magnetic rack for 1 min.
29. Aspirate and discard the supernatant. Remove the tube from the magnetic rack and resuspend the magnetic beads in 80 μL of depletion buffer (DB). Place the tube back on the magnetic rack for 1 min. Aspirate and discard the supernatant.
30. Add 80 μL of DB to the magnetic beads and mix by pipetting. Do not place the tube back on the magnetic rack.

#### rRNA depletion

31. Once your sample has cooled, add the 20 μL of sample into your magnetic bead suspension and pipette up and down several times to mix. Let sit at 20°C–25°C for 5 min. Place onto a thermomixer set to 37°C for 5 min. Skip the 50°C incubation step.
32. Spin down droplets and place the tube on a magnetic rack for 2 min. Transfer the supernatant (∼85–90 μL) to a new tube.
33. Adjust the volume of each sample to 100 μL with nuclease-free water and proceed to cleaning RPF RNAs using the Zymo RNA Clean and Concentrator kit.
  a. Add 200 μL RNA binding buffer and 450 μL 100% ethanol to each RPF sample.
  b. Load each column with the RPF sample, spin for 1 min at >10,000 × *g* and discard the flow-through.
  c. Wash each column with 400 μL RNA prep buffer and spin at >10,0000 × *g* for 1 min and discard the flow-through.
  d. Wash each column with 700 μL RNA wash buffer and spin at >10,000 × *g* for 1 min and discard the flow-though.
  e. Wash each column with 400 μL RNA wash buffer and spin at >10,000 × *g* for 2 min and discard the flow-through. Transfer each column to a fresh, nuclease-free centrifuge tube.
  f. Elute RPF RNA samples with 11 μL nuclease-free water and keep the samples on ice or store at −20°C for up to a week. Expect to recover ∼ 10 μL.

### PAGE purification of ribosome-protected fragments (RPFs)

**Timing: 3 h and 30 min**

RPF fragments corresponding to lengths between 27 and 30 nt are extracted from a denaturing polyacrylamide gel.

34. Prepare the RPF sample(s), 20/100 oligo ladder, and RNA control marker (27, 30 nt) by mixing the following materials.
  a. Add 10 μL of 2× TBE-urea dye to each RPF sample.
  b. 20/100 oligo ladder: 1 μL 20/100 oligo ladder (0.1 μg/μL), 19 μL nuclease-free water, 20 μL 2× TBE-urea dye.
  c. RNA control marker: 1 μL 27 nt marker (10 μM), 1 μL 30 nt marker (10 μM), 18 μL nuclease-free water.
35. Denature the samples, ladder, and RNA control marker by boiling them at 95°C for 5 min. Place the samples on ice immediately after.
36. Prepare 800 mL of fresh 1× TBE buffer to run the gel.
37. Prepare a 15% TBE-urea polyacrylamide gel by rinsing the wells with 1× TBE buffer and pre-running the gel for 5 min at 180 V prior to loading the samples.
38. Load 10 μL of the 20/100 oligo ladder, 10 μL of the marker, and 20 μL of each sample across two lanes (10 μL per lane).

***Note:*** It is recommended to load markers next to each sample to help with cutting the gel.

**Figure 2:**
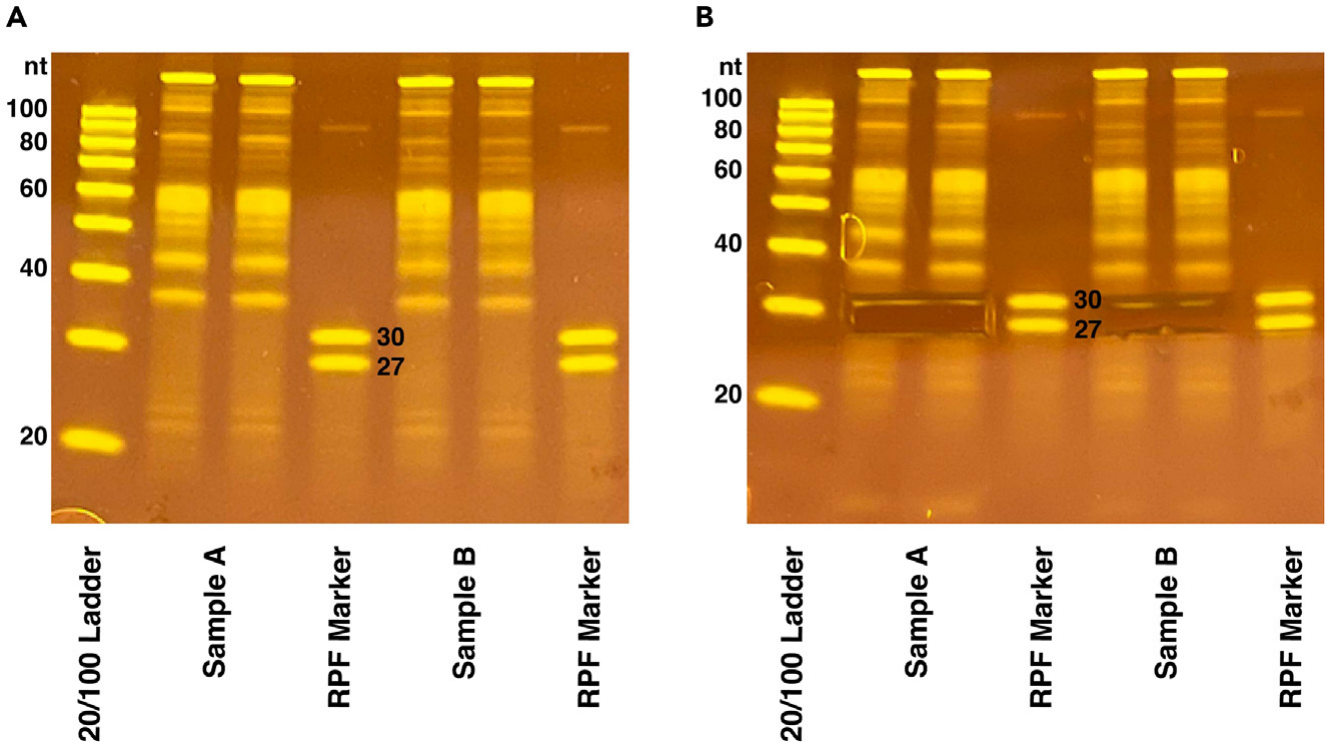
15% TBE-Urea Gel (A) Example RPF gel showing digested RNA post-rRNA depletion. 20 μL of sample is loaded across two lanes. (B) RPF gel with 27–30 nt regions cut out for Ribo-seq samples.
43. Centrifuge the tube(s) at >15,000 × *g* for 2 min to shred the gel slices. Gel pieces should all be in the 1.5 mL microcentrifuge tube.

***Note:*** The gel slice may be cut up further into smaller pieces before centrifuging if you find that it does not pass through the 18-gauge hole in the 0.5 mL tube easily.

39. Run the gel at 180 V for 65 min or until the bromophenol blue bands reach the bottom of the gel.
40. Stain each gel with 3 μL of SYBR gold and 30 mL of 1× TBE buffer on a shaker at 20°C–25°C for 20 min.
41. Create a hole in the bottom of a 0.5 mL tube using an 18-gauge needle. Place that 0.5 mL tube in a 1.5 mL tube. This will be used for shredding the gel.
42. Using the 27 and 30 nt marker bands on the gel as a reference, cut the 27–30 nt region for each sample and place the excised bands in a 0.5 mL tube that was prepared in the previous step (Figure 2).

44. Remove and discard the 0.5 mL tube.
45. Add the following components to each 1.5 mL microcentrifuge tube with gel pieces and rotate the samples for 2 h at 20°C–25°C to extract RPFs.
  a. 400 μL nuclease-free water.
  b. 40 μL 5 M ammonium acetate.
  c. 2 μL 10% sodium dodecyl sulfate (SDS).
  d. 1 μL Rnase inhibitor (Superasin).
46. Trim the end of a 1 mL pipette tip with a fresh razor and use that tip to transfer the gel slurry to a centrifugal filter. Centrifuge the tube(s) at 2,000 × *g* for 3 min.
47. Transfer the RNA solution to a fresh 1.5 mL microcentrifuge tube and add the following reagents to each tube.
  a. 2 μL Glycoblue.
  b. 700 μL 100% isopropanol.
48. Mix well and incubate for >2 h at −20°C.
49. Centrifuge the tube(s) at 15,000 × *g* at 4°C for 20 min to pellet the RPF RNA.
50. Decant the supernatant without disturbing the pellet.
51. Wash the pellet with 500 μL of ice-cold 80% ethanol and centrifuge at 15,000 × *g* at 4°C for 10 min.
52. Decant the supernatant without disturbing the pellet and air dry for about 10 min.
53. Resuspend the pellet in 3.5 μL of nuclease-free water.

**Pause point:** Sample(s) can be stored at −20°C for up to a week.

### Pre-adenylation of adapter

**Timing: 1 h and 30 min**

Prior to the ligation of the adapter to the RPF, the 5′ end of the 3′ adapter is adenylated to allow for ligation with RNA ligase.

***Note:*** In this protocol, we use the 3′ adapter sequence from McGlincy et al.,^10^ but do not include the barcode sequence.

54. Combine the following components in a tube.

**Table:** Adapter Adenylation

Reagent	Amount
100 μM 3′ adapter	1.2 μL
10× 5′ DNA adenylation reaction buffer	2 μL
1 mM ATP	2 μL
Nuclease-free water	12.8 μL
Mth RNA Ligase	2 μL
Total	20 μL

55. Incubate the tube at 65°C for 1 h followed by heat-inactivation of the enzyme at 85°C for 5 min.
56. Adjust the volume of each sample to 50 μL by adding 30 μL of nuclease-free water. Purify the sample(s) with the Zymo RNA Clean and Concentrator kit.
  a. Add 100 μL RNA binding buffer and 150 μL 100% ethanol.
  b. Load the column with the sample, spin for 1 min at >10,000 × *g*, and discard the flow-through.
  c. Wash the column with 400 μL RNA prep buffer and spin at >10,0000 × *g* for 1 min, and discard the flow-through.
  d. Wash the column with 700 μL RNA wash buffer and spin at >10,000 × *g* for 1 min, and discard the flow-though.
  e. Wash the column with 400 μL RNA wash buffer and spin at >10,000 × *g* for 2 min, and discard the flow-through. Transfer the column to a fresh, nuclease-free centrifuge tube.
  f. Elute with 7 μL nuclease-free water and keep the sample on ice or store at −20°C for up to a month. Expect to recover ∼6 μL.
57. Store the adenylated adapter at −20°C.

***Note:*** Avoid repeated freeze-thaw of the adenylated adapter. Make aliquots prior to storing in the −20°C. Adapters should be prepared fresh after one month of storage at −20°C.

### End repair of RPFs

**Timing: 1 h**

The 3′ phosphate at the end of each RPF is removed to allow for ligation with the adenylated 3′ adapter.

58. Add the following to the 3.5 μL RPF samples. Do not make a master mix and add each component individually.

**Table:** End repair reagents

Reagent	Amount
10× T4 PNK buffer	0.5 μL
Promega RNasin Plus inhibitor	0.5 μL
T4 PNK enzyme	0.5 μL

59. Mix well and incubate the samples at 37°C for 1 h.

**Pause point:** Sample(s) can be stored at −20°C for up to a week.

### Adapter ligation

**Timing: 5 h and 15 min**

The end-repaired RPFs are ligated to the adenylated 3′ adapter. Leftover adapters are then removed using deadenylase and exonuclease.

60. Add 1 μL of adenylated adapter to each end repaired RPF sample and heat denature the sample(s) at 65°C for 2 min.

***Note:*** Hold the temperature at 4°C immediately after heat denaturation.

61. Add the following components to each heat-denatured sample. Do not make a master mix and add each component individually.

**Table:** Adapter Ligation Components

Reagent	Amount
10× T4 RNA Ligation Buffer	1 μL
40% PEG-8000 (∼17% final)	6.5 μL
T4 RNA Ligase 2	1 μL

***Note:*** Allow 40% PEG-8000 to equilibrate at 20°C–25°C for about 20 min prior to adding it to the samples.

62. Centrifuge briefly to consolidate the sample at the bottom of the tube(s).
63. Incubate the sample(s) at 23°C for 3 h.

**Pause point:** Sample(s) can be stored at −20°C.

64. Add 1 μL of RecJ Exonuclease and 0.5 μL of Deadenylase to each sample and mix thoroughly by pipetting.
65. Incubate the samples at 30°C for 2 h.

**Pause point:** Sample(s) can be stored at −20°C

### Reverse transcription

**Timing: 1 h and 30 min**

Convert RNA samples into single-stranded cDNA using a reverse transcription (RT) primer that recognizes complementary sequences on the adapter-ligated to the RPFs in the previous step. Following reverse transcription, the RNA template is degraded.

***Note:*** We use the reverse transcription primer from McGlincy et al.,^10^ but do not include the first two random nucleotide sequences.

66. Prepare the following mix for each sample:

**Table:** Reverse Transcription mix

Reagent	Amount
Promega RNasin Plus inhibitor	0.5 μL
10 mM dNTP mix	1 μL
10× Reverse Transcription (RT) buffer	3 μL
100 mM DTT	1.5 μL
5 μM RT primer	1 μL
EpiScript Reverse Transcriptase	1 μL
Nuclease-free water	5 μL
Total Volume	13 μL

***Note:*** Do not create a master mix. Prepare each mix individually per sample.

67. Add the 13 μL reverse transcription mix to each sample and mix well by pipetting.
68. Incubate the sample(s) at 50°C for 30 min.
69. Add 1 μL of Exonuclease I to each sample and incubate at 37°C for 30 min followed by 15 min of incubation at 80°C. Hold samples at 4°C or place the samples on ice.

**Pause point:** Sample(s) can be stored at −20°C

70. Add 1 μL Hybridase to each sample and incubate at 55°C for 5 min and hold at 4°C or place the samples immediately on ice.
71. The volume should now be 31 μL. Adjust the volume of each sample to 50 μL by adding 19 μL of nuclease-free water. Purify the samples with the Zymo RNA clean and concentrator kit.
  a. add 100 μL RNA binding buffer and 150 μL 100% ethanol.
  b. Load each column with the sample(s), spin for 1 min at >10,000 × *g* and discard the flow-through.
  c. Wash each column with 400 μL RNA prep buffer and spin at >10,0000 × *g* for 1 min and discard the flow-through.
  d. Wash each column with 700 μL RNA wash buffer and spin at >10,000 × *g* for 1 min and discard the flow-though.
  e. Wash each column with 400 μL RNA wash buffer and spin at >10,000 × *g* for 2 min and discard the flow-through. Transfer each column to a fresh, nuclease-free centrifuge tube.
  f. Elute cDNA samples with 11 μL nuclease-free water and keep the samples on ice or store at them −20°C. Expect to recover ∼ 10 μL.

**Pause point:** Sample(s) can be stored at −20°C.

### PAGE purification cDNA

**Timing: 3 h and 30 min**

cDNA fragments are extracted from a denaturing polyacrylamide gel to separate from potential side reaction products.

72. Prepare the 20/100 oligo ladder and sample(s):
  a. Add 10 μL 2× TBE-urea dye to each sample.
  b. 20/100 oligo ladder: 1 μL 20/100 oligo ladder (0.1 μg/μL), 19 μL nuclease free water, 20 μL 2× TBE-urea dye.
73. Denature the samples and ladder by boiling them at 95°C for 5 min. Place the sample(s) on ice immediately after.
74. Prepare 800 mL of fresh 1× TBE buffer to run the gel.
75. Prepare a 10% TBE-urea polyacrylamide gel by rinsing the wells with 1× TBE buffer and pre-running the gel for 5 min at 180 V prior to loading the sample(s).
76. Load 10 μL of the 20/100 oligo ladder or 20 μL of sample per lane.
77. Run the gel at 180 V for 65 min or until the bromophenol blue bands reach the bottom of the gel.
78. Stain the gel with 3 μL of SYBR gold and 30 mL of 1× TBE running buffer on a shaker at 20°C–25°C for 20 min.
79. Create a hole in the bottom of a 0.5 mL tube using an 18-gauge needle. Place that 0.5 mL tube in a 1.5 mL tube. This will be used for shredding the gel.
80. Cut out the 85–105 nt bands using a fresh razor blade (Figure 3).
  ***Note:*** a band at this size range may not be visible due to the low amount of cDNA, but successful libraries can sometimes still be made in these cases.

**Figure 3:**
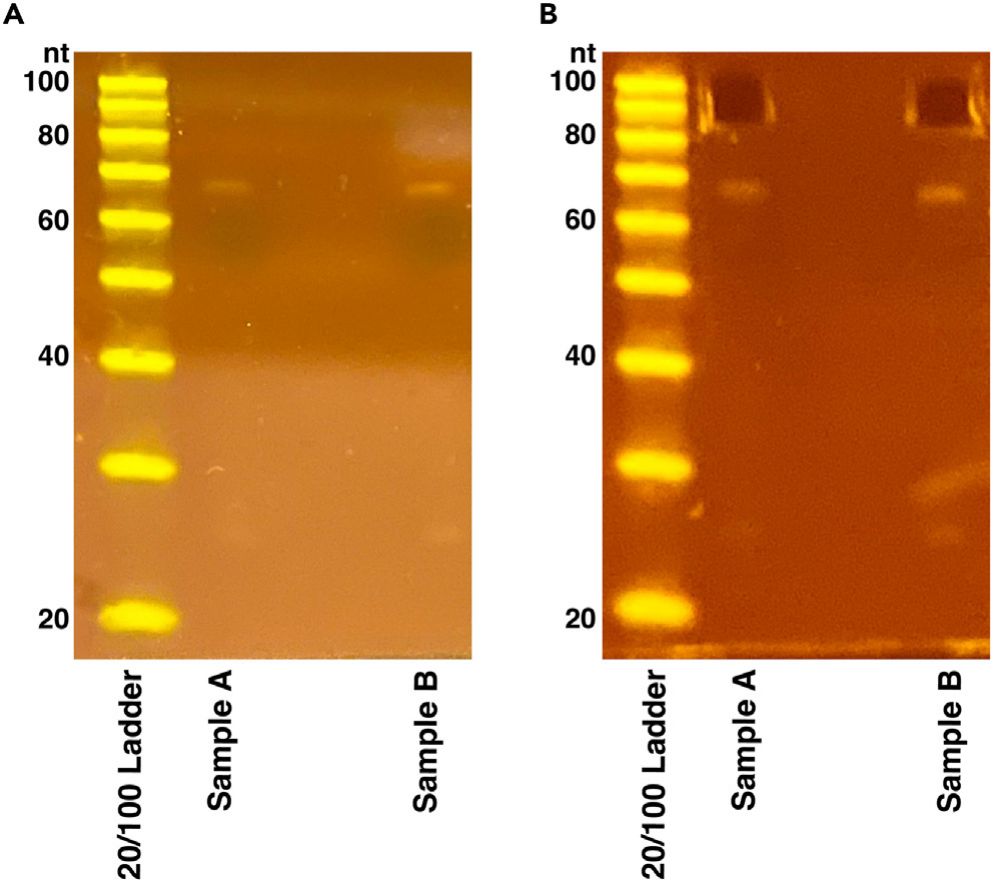
10% TBE Urea Gel (A) Example cDNA gel. 20 μL of sample is loaded into one lane. Proceed with gel extraction regardless of whether a band is visible between 85–105 nt. (B) cDNA gel after extraction showing region cut. ***Note:*** The band near 70 nt corresponds to no insert reverse transcription products.
81. Transfer the slice(s) to the 0.5 mL tube(s) and centrifuge at 14,000 × *g* for 2 min.
82. Remove and discard the 0.5 mL tube(s).
83. To each sample add the following components:
  a. 400 μL nuclease-free water.
  b. 40 μL 5 M ammonium acetate.
  c. 2 μL 10% SDS.
84. Incubate the sample(s) in a thermomixer at 37°C for 2 h at 900 rpm.
85. Trim the end of a 1-mL pipette tip with a fresh razor and use that tip to transfer the gel slurry to a 1.5 mL centrifugal filter. Centrifuge the tube(s) at 2,000 × *g* for 3 min.
86. Transfer the cDNA solution to a fresh 1.5 mL microcentrifuge tube and add the following to each tube.
  a. 2 μL Glycoblue.
  b. 700 μL 100% isopropanol.
87. Mix well and incubate the sample(s) for >2 h at −20°C.
88. Centrifuge the tube(s) at >12,000 × *g* (4°C) for 20 min to pellet the cDNA.
89. Decant the supernatant without disturbing the pellet.
90. Wash the pellet with 500 μL of ice-cold 80% ethanol and centrifuge (4°C) at >12,000 × *g* for 10 min.
91. Decant the supernatant without disturbing the pellet and air dry for about 10 min.
92. Resuspend the pellet in 10 μL of nuclease-free water.

**Pause point:** Store the sample(s) at −20°C.

### cDNA circularization

**Timing: 2 h**

Circularize cDNA to flank both sides of the RPF cDNA with primer binding sites for PCR amplification.

93. Prepare the following CircLigase mix for each sample:

**Table:** CircLigase mix

Reagent	Amount
10× Circligase reaction buffer	2 μL
1 mM ATP	1 μL
50 mM MnCl_2_	1 μL
CircLigase	1 μL
Nuclease-free water	5 μL
Total	10 μL

***Note:*** Do not create a master mix. Prepare each mix individually per sample.

94. Add the 10 μL CircLigase mix to each sample and mix by pipetting. Centrifuge the sample(s) briefly.
95. Incubate the sample(s) at 60°C for 2 h. Next, hold the samples at 4°C or immediately place them on ice.

**Pause point:** Sample(s) can be stored at −20°C

### PCR amplification of cDNA

**Timing: 50 min**

PCR amplify the circularized cDNA products to yield a dsDNA RPF library that is ready for Illumina sequencing.

***Note:*** Reverse primer sequences used at this step in the protocol are from the now discontinued Illumina TruSeq Ribo Profile kit.

96. Pick a unique index primer for each sample while considering the recommended combinations for optimal index color balancing.

**Table:** 

Primer	Index sequence
Index 1 PCR primer	5′-CGTGAT-3′
Index 2 PCR primer	5′-ACATCG-3′
Index 3 PCR primer	5′-GCCTAA-3′
Index 4 PCR primer	5′-TGGTCA-3′
Index 5 PCR primer	5′-CACTGT-3′
Index 6 PCR primer	5′-ATTGGC-3′
Index 7 PCR primer	5′-GATCTG-3′
Index 8 PCR primer	5′-TCAAGT-3′
Index 9 PCR primer	5′-CTGATC-3′
Index 10 PCR primer	5′-AAGCTA-3′
Index 11 PCR primer	5′-GTAGCC-3′
Index 12 PCR primer	5′-TACAAG-3′

**Table:** 

Duplex (two indices)	Index 06 and index 12	Perfect base color balance
Triplex (three indices)	Index 04 and Index 08	add index 01, 02, 05, 06, 09, or 10
Triplex (three indices)	Index 07 and Index 11	add index 01, 02, 05, 06, 09, or 10
Triplex (three indices)	Index 02 and Index 09	add index 03, 04, 07, 08, 11, or 12
Four or more indices	Index 06 and Index 12	add any other indices

97. Prepare the following PCR mix for each sample.

**Table:** PCR mix

Reagent	Amount
Nuclease-free water	16 μL
Ribo PCR Forward Primer (10 μM)	2 μL
Ribo PCR Reverse Index primer (10 μM)	2 μL
2× Phusion Hot Start master mix	25 μL
Total	45 μL

***Note:*** Do not create a master mix, prepare each mix individually per sample.

98. Add 5 μL of the cDNA to the PCR master mix and mix thoroughly.
99. Place the tubes in a thermocycler and use the following program:

**Table:** PCR cycling conditions

Steps	Temperature	Time	Cycles
Initial Denaturation	98°C	30 s	1
Denaturation	94°C	15 s	11–15 cycles
Annealing	55°C	5 s	
Extension	65°C	10 s	
Hold	4°C	∞

***Note:*** The number of PCR cycles can be raised to a maximum of 15 cycles.

100. Clean up PCR products with 1.8× Agencourt AMPure XP beads.
101. Aliquot 90 μL of the beads for each sample and allow them to reach 20°C–25°C.
102. Mix the PCR products with the beads thoroughly by pipetting at least 10 times. Incubate the bead and PCR mixture for 5 min at 20°C–25°C.
103. Place the tube(s) on a magnetic rack and wait 5 min.
104. While keeping the tube(s) on the magnetic rack, remove the supernatant and add 200 μL of 80% ethanol to each tube. Wait 30 s and remove the ethanol.
105. Repeat the ethanol wash for a total of two ethanol washes.
106. Air dry the beads for up to 2 min until the ethanol in the tube evaporates.

***Note:*** Avoid over drying the beads as this may decrease the efficiency of the elution.

107. Remove the tube(s) from the magnetic rack and resuspend the beads in 25 μL of nuclease-free water.
108. Incubate the tube(s) at 20°C–25°C for 2 min.
109. Place the tube(s) back on the magnetic rack and wait 1 min.
110. Carefully pipette the PCR products out of the tube(s) and transfer them to fresh tube(s).
111. Measure the DNA concentration of each sample using the dsDNA HS Qubit assay kit.
112. Submit the sample(s) for BioAnalyzer quality control.

**Figure 4:**
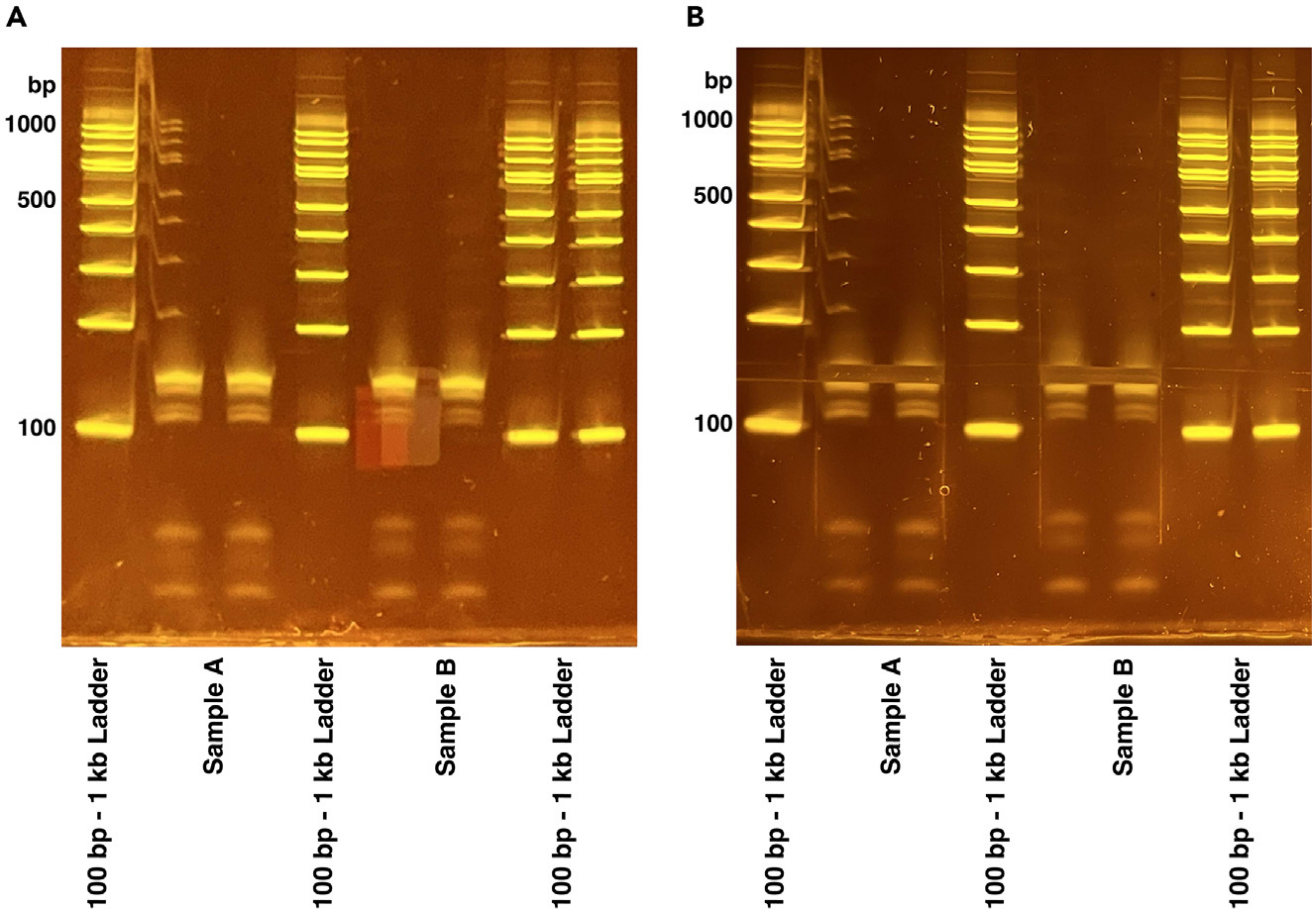
8% TBE Gel (A) Example final library PCR product gel. 60 μL of sample is loaded across two lanes. RPF library migrates between 100 and 200 bp. (B) PCR gel after extraction showing region cut.

**Figure 5:**
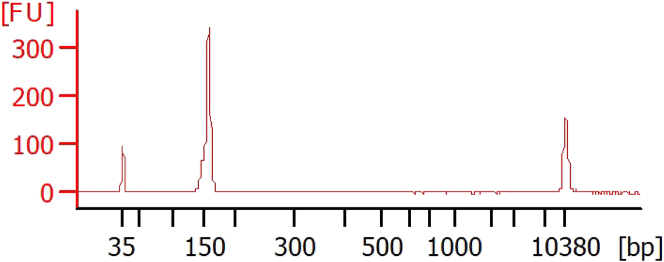
Bioanalyzer profile of RPF library after PCR amplification and gel extraction cleanup The final RPF library is 150–160 bp and is a clean single peak as expected.

**Pause point:** Sample(s) can be stored at −20°C

### PAGE purification of PCR products

**Timing: 3 h**

The final dsDNA RPF library is extracted from a non-denaturing polyacrylamide gel to separate the library from unwanted side products such as adapter dimers (troubleshooting 5). If the library is already a clean single peak of the correct library size, then skip this step and proceed to sequencing.

113. Prepare the sample(s) and ladder:
  a. Add 6 μL of 5× native TBE loading dye to each 25 μL of PCR products.
  b. Use 6 μL of the 100 bp–1 kb ladder.
114. Prepare 800 mL of 1× TBE running buffer to run the gel.
115. Prepare an 8% native TBE polyacrylamide gel by rinsing the wells with 1× TBE buffer and pre-running the gel for 5 min at 180 V before loading the sample(s).
116. Load 30 μL of each sample across two lanes (15 μL per lane) or load 6 μL of ladder in one lane.
117. Run the gel at 200 V for 40 min.
118. Stain the gel with 3 μL of SYBR gold and 10 mL of 1× TBE running buffer on a shaker at 20°C–25°C for 20 min.
119. Create a hole in the bottom of a 0.5 mL tube using an 18-gauge needle. Place that 0.5 mL tube in a 1.5 mL tube. This will be used for shredding the gel.
120. Using a fresh razor blade, cut out the band at 140–160 bp and transfer it to the 0.5 mL tube(s) (Figure 4).
121. Centrifuge the tube(s) at 14,000 × *g* for 2 min. Discard the 0.5 mL tube(s) that was used for shredding the gel.
122. To each 1.5 mL tube, add the following components:
  a. 400 μL nuclease-free water.
  b. 40 μL 5 M ammonium acetate.
  c. 2 μL 10% SDS.
123. Incubate the sample(s) on a thermomixer at 37°C (900 rpm) for 2 h.
124. Trim the end of a 1-mL pipette tip with a fresh razor and use that tip to transfer the gel slurry to a 1.5 mL centrifugal filter. Centrifuge the tube(s) at 2,000 × *g* for 3 min.
125. Transfer the PCR product solution to a fresh 1.5 mL microcentrifuge tube and add the following to each tube:
  a. 2 μL Glycogen.
  b. 700 μL 100% isopropanol.
126. Mix each tube well and incubate >2 h at −20°C.
127. Centrifuge the tube(s) at >12,000 × *g* (4°C) for 20 min to pellet the PCR products.
128. Decant the supernatant without disturbing the pellet.
129. Wash the pellet with 500 μL of ice-cold 80% ethanol and centrifuge (4°C) at >12,000 × *g* for 10 min.
130. Decant the supernatant without disturbing the pellet and air dry for about 10 min.
131. Resuspend the pellet in 25 μL of nuclease-free water.
132. Measure the concentration of PCR products using the Qubit dsDNA high-sensitivity kit.
133. Submit samples for BioAnalyzer quality control and sequencing (Figure 5).

**Pause point:** Sample(s) can be stored at −20°C

### Processing ribosome profiling sequencing data

**Timing: 4–5 h**

We use an in-house integrated pipeline including RibORF 0.1, a software package used to score ORFs for translation based on Ribo-seq coverage. There are other ORF prediction tools that are newer than RibORF 0.1 that may also be used in addition or instead of RibORF.

***Note:*** It is recommended that users create a conda environment to manage the specific dependencies and tools needed to run the workflow. The pipeline will run optimally in a high-performance computing cluster environment. Users can create a conda environment using the following commands after installation of Anaconda or Miniconda.

conda create -name Riboseq_env python=2.7.18

conda activate Riboseq_env

conda install -c bioconda star

conda install -c bioconda homer

conda install -c bioconda fastx_toolkit

conda install -c bioconda samtools

conda install -c bioconda bedtools

134. Obtain FASTQ files from the sequencer.

***Note:*** If the sample is paired end, then proceed with processing with the read 1 fastq file. The length of time for processing the sequencing files will depend on the size of the fastq file.

135. Install the required software tools of the pipeline and load the conda environment (see materials list above).
136. Download the fasta files for your reference genome. We will use the mouse mm10 reference genome for our example (https://hgdownload.soe.ucsc.edu/goldenPath/mm10/chromosomes/).

***Note:*** We only include primary chromosomes in our alignments.

137. Download the gtf file for the reference annotation for your species. We will use the mouse GENCODE annotation in our example, which can be downloaded from the GENCODE website (https://www.gencodegenes.org/mouse/).
138. Download the fasta files for the rRNA and tRNA sequences using the UCSC genome browser’s Table Browser tool to pull these sequences from the Repeat Masker tracks.
139. Download the RefSeq gene refFlat file and create a new file including only the coding genes annotated as “NM”. In our case we used the mouse mm10 version (http://hgdownload.cse.ucsc.edu/goldenPath/mm10/database/).

***Note:*** Newer reference annotations may be used as available.

140. Generate the STAR index for both the reference genome annotation and the tRNA and rRNA files.

STAR --runMode genomeGenerate --genomeDir Reference output directory (mm10.star) --genomeFastaFiles input fasta --sjdbGTFfile gencode.gtf

STAR --runMode genomeGenerate --genomeDir tRNA_rRNA output directory (mm10cont.star) --genomeFastaFiles tRNA_rRNA fasta --genomeSAindexNbases 7

***Note:*** A *de novo* transcriptome assembly can be used as an option in place of a reference annotation if there are RNA-seq samples sequenced in parallel with Ribo-seq data.

141. For Ribo-seq fastq processing, the first step is to trim the 3′ adapter sequence using the FASTX-toolkit. Untrimmed reads and reads less than 20 nt after trimming are discarded.

zcat Sample.fastq.gz | fastx_clipper -Q33 -l 20 -n -v -c -a AGATCGGAAGAGCACACGTCTGAAC | fastx_trimmer -Q33 -f 1 2>> Sample.trimlog > Sample_trimmed_R1.fastq

pigz Sample_trimmed_R1.fastq

142. The trimmed reads are then aligned to rRNA and tRNA genes, which represent unwanted contaminants in Ribo-seq samples. Reads that align to these regions are discarded and the remaining reads are aligned to the entire genome. In these alignments we allow for multimapping reads for user flexibility, but later on we will filter out mutlimappers and use only uniquely aligned reads in our analyses.

***Note:*** STAR is our preferred splice-aware read alignment tool, but other similar tools can be substituted.

STAR --readFilesCommand zcat --outSAMstrandField intronMotif --outReadsUnmapped Fastx --genomeDir mm10cont.star --runThreadN 16 --readFilesIn Sample_trimmed_R1.fastq.gz --outFileNamePrefix Sample_trimmed_cont

STAR --outSAMstrandField intronMotif --genomeDir mm10.star --runThreadN 16 --readFilesIn Sample_trimmed_contUnmapped.out.mate1 --outFileNamePrefix Sample_trimmed_filtered --outFilterMismatchNmax 2 --outFilterMultimapNmax 4 --chimScoreSeparation 10 --chimScoreMin 20 --chimSegmentMin 15 --outSAMattributes All --outSAMtype BAM SortedByCoordinate

143. The resulting bam file Sample_trimmed_filteredAligned.sortedByCoord.out.bam is filtered for primary alignments using samtools to keep only the best scoring aligned multimappers.

samtools view -bS -F 0X100 Sample_trimmed_filteredAligned.sortedByCoord.out.bam > Sample_trimmed_filteredAligned.sorted.noSecondaryAlign.bam

144. The resulting .bam file is used to generate bedgraphs through HOMER that can be uploaded and visualized in the UCSC genome browser or Integrative Genomics Viewer to look at Ribo-seq coverage. There are two bedgraph files that are generated. The Sample_trimmed_filtered_noSecondaryAlign.bedGraph file includes all reads from the .bam generated in step 143 including multimappers. The Sample_trimmed_filtered_noMulitMap.bedgraph file includes only the uniquely aligned reads.

makeTagDirectory tags_multi/Sample_trimmed_filtered_noSecondaryAlign Sample_trimmed_filteredAligned.sorted.noSecondaryAlign.bam -keepAll -totalReads all

makeUCSCfile tags_multi/Sample_trimmed_filtered_noSecondaryAlign -o Sample_trimmed_filtered.noSecondaryAlign.bedGraph -fragLength given -strand separate

makeTagDirectory tags_nomulti/Sample_trimmed_filtered_noMultiMap Sample_trimmed_filteredAligned.sorted.noSecondaryAlign.bam

makeUCSCfile tags_nomulti/Sample_trimmed_filtered_noMultiMap -o Sample_trimmed_filtered.noMultimap.bedGraph -fragLength given -strand separate

145. After generating the bedgraph files, samtools is then used to remove the multimappers from the read file generated in step 143. This creates a read alignment file with only uniquely aligned reads and is used in the subsequent steps. Users can also choose to use the read alignment file that retains multimappers.

samtools view -bq 255 Sample.sorted.noSecondaryAlign.bam > Sample.sorted.noMultiMap.bam

**Figure 6:**
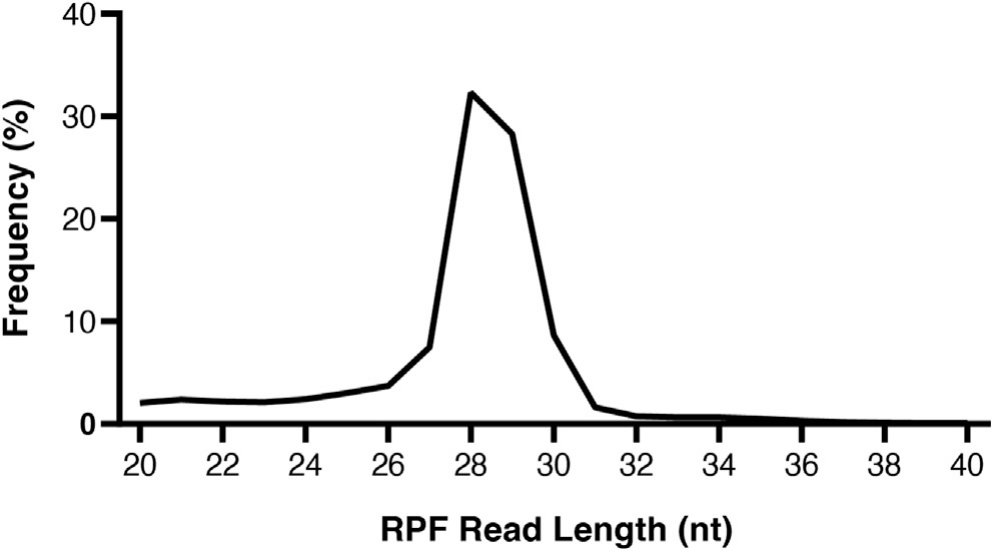
Length distribution plot of RPF reads RPF lengths peak around 28–29 nt as expected for fully digested ribosome footprints.

***Note:*** High quality Ribo-seq data should have the majority of the reads centered around 28–29 nt read lengths. You can plot the length frequency values in a graphing software tool such as R, Excel, or Prism to visualize the frequency distribution of the reads.

146. Determine the RPF read length distribution (Figure 6). One million reads are randomly sampled from the bam file and sorted by the read length.

samtools view Sample.sorted.noMultiMap.bam | grep -E '(nM:i:0)|(ˆ@)'|awk -v v1=0.05 'BEGIN {srand()} !/ˆ$/ { if (rand() <= v1) print length($10)}' | head -n 1000000 | sort | uniq -c > Sample_alignedLengthHistogram.txt

147. Visualize the aligned RPFs of each length relative to annotated CDS regions to choose the best offset correction such that the 5′ end of reads are aligned to ribosomal A-sites within coding regions.

***Note:*** A-site reads are defined by the first position of the codon located in the A-site of the RPF. For this example, we use the RefSeq coding gene refFlat file for our annotated coding regions. First, the .bam file from step 145 is converted to a .sam file using samtools. The pipeline will take the .sam file and run the readDist.pl script from the RibORF 0.1 software package to generate metagene plots to show the average read distributions from 30 nt upstream to 50 nt downstream of start sites and vice versa for stop sites. The step will generate plots for a given read length that the user selects. In the example below, an uncorrected metagene plot is generated for 30 nt reads. Repeat this step for all read lengths across the length distribution peak. For our example in Figure 6, we included read lengths 26–31 nt for visualization of metagenes.

samtools view -h Sample.sorted.noMultiMap.bam > Sample.sorted.noMultiMap.sam

perl RibORF_v0.1/readDist.pl Sample.sorted.noMultiMap.sam mm10.RefSeq.coding.refFlat.txt Output File 30 30 50

148. Choose the read lengths to include for offset correction based on the metagene plots generated in step 147. Only read lengths with good 3 nt periodicity should be included for offset correction. These will be the final set of reads used in smORF translation scoring.

***Note:*** During offset correct each, the number of bases to be shifted such that the 5′ end of the read aligns with the first position of the A-site codon is determined. For example, when analyzing high-resolution datasets, 28 nt foot prints usually require a 15 nt offset to align with the A-site. An offset parameters text file will need to be created as input into the offsetCorrect.pl script. The offset parameters file needs to be formatted into two columns separated by a tab. The first column is the read lengths to be offset corrected and the second column is the offset distance.

perl offsetCorrect.pl Sample.sorted.noMultiMap.sam Sample.offset.correction.parameters.txt Sample.corrected.sam

**Figure 7:**
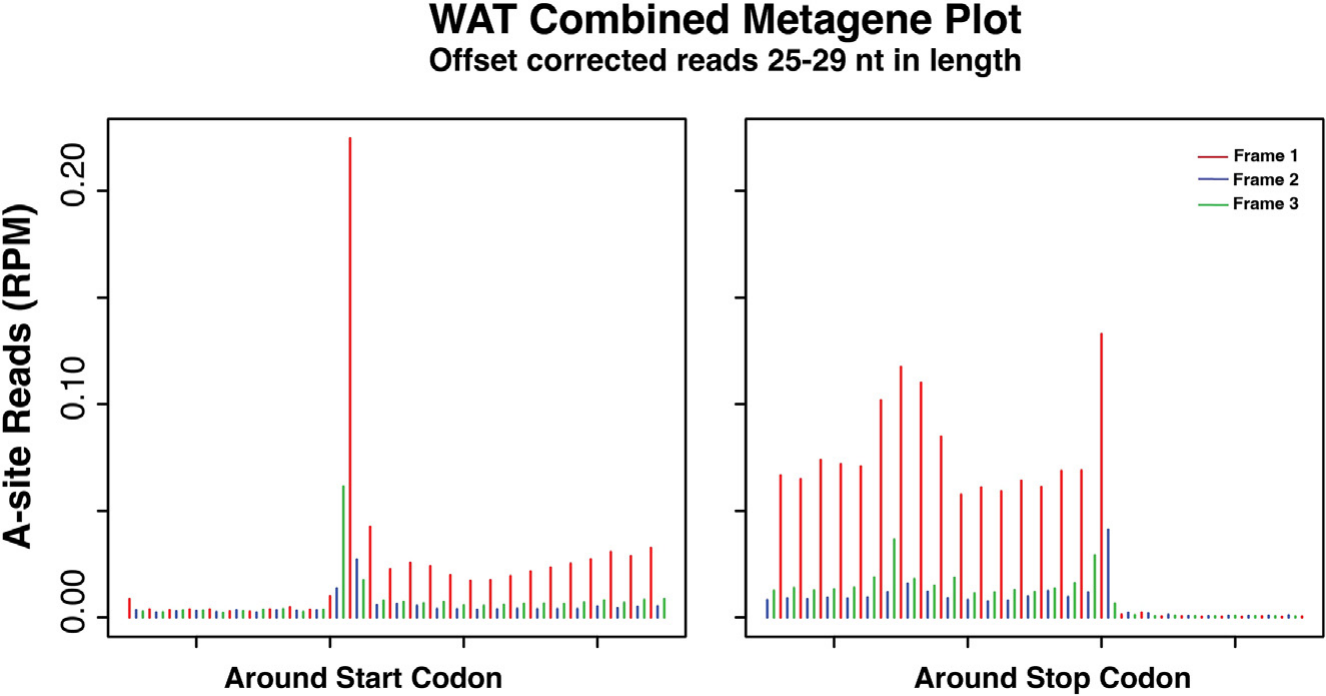
Combined metagene plot of ribosome A-site offset corrected RPF reads Metagene plot shows good coverage and 3 nt periodicity within the translation initiation and termination sites. The different colored bars represent the three possible reading frames for translation. Red is reading frame 1 and is the coding frame for our metagene, blue is frame 2, and green is frame 3. RPM, reads per million.

149. Run the readDist.pl script again using the corrected.sam file generated in step 148 to plot the corrected read locations of all lengths chosen by the user (Figure 7). The read length should be set to 1 as all reads now represent the first position of the ribosomal A-site.

perl readDist.pl Sample_corrected.sam hg38.RefSeq.coding.refFlat.txt outputDirectory 1 30 50

150. Translate the reference transcriptome in all three reading frames using the GTFtoFasta Java script.

***Note:*** This script will parse all possible open reading frames, finding the most upstream canonical start codon and an in frame stop. If there is no canonical start codon, then the ORF will be defined by stop site to stop site. The output contains a .gtf file of all possible ORFs as well as peptide and nucleotide sequences for each ORF. Usage of the GTFtoFasta script is shown below using the GENCODE reference gtf.

java GTFtoFasta gencode.annotation.gtf mm10.fa [MsOnly]

151. Use the gtfToGenePred script from the UCSC tool collection to convert the ORFs gtf file generated in the previous step to a genePred file format. A custom python script is then used to convert the resulting genePred file to refFlat format.

gtfToGenePred gencode.gtf gencode.gtf.genePred

python2 genePred-to-refFlat-fixCoord.py gencode.gtf.genePred gencode.refFlat

***Note:*** The genePred-to-refFlat-fixCoord.py script shown below reformats the genePred to a refFlat format.

import sys

inputfile1 = sys.argv[1]

outputfile1 = sys.argv[2]

infile1 = open(inputfile1,'rU')

outfile1 = open(outputfile1,'w')

for i in infile1:

 split1 = i.strip().split('∖t')

 outfile1.write(split1[0]+'∖t'+'.'+'∖t'+ split1[1]+'∖t'+ split1[2]+'∖t'+ split1[3]+'∖t'+ split1[4]+'∖t')

 split[5] = split1[3]

 outfile1.write(split1[5]+'∖t'+ split1[6]+'∖t'+ split1[7]+'∖t'+ split1[8]+'∖t'+ split1[9]+'∖n')

infile1.close()

outfile1.close()

152. Run the RibORF.pl script to score each ORF for translation using the offset corrected .sam file from step 148 and the ORF refFlat file from the previous step as inputs.

***Note:*** The R package required to be used with RibORF 0.1, e1071, is a support vector machine classifier that needs to be installed in the user’s PATH to run RibORF.pl. The resulting output file of predicted ORFs is the pred.pvalue.parameters.txt. We use an ORF length cutoff of 12 nt and a minimum read coverage cutoff of 10 reads.

perl ribORF.pl -Sample_corrected.sam ORFs.refFlat outputDirectory 12 10

153. Filter the ORF list for predicted translated smORFs by selecting for candidates that have a minimum p-value cutoff of 0.7 (based on the RibORF authors’ suggestion), maximum nucleotide length cutoff of 450 (representing ORFs of 150 codons and smaller), and a minimum read coverage cutoff of 10.

awk '{ if ($15 >= 0.7 && $7 <= 450 && $8 >= 10) print $0 }' FS='∖t' OFS='∖t' pred.pvalue.parameters.txt > pred.pvalue.parameters.filtered.txt

***Note:*** The output pred.pvalue.parameters.filtered.txt file from step 153 is the list of smORFs that are predicted by RibORF to be translated. There are additional filtering steps that can be applied to remove smORFs that are already annotated or overlap with known coding genes. Additional tools that are recommended to be used are bedtools intersect and NCBI blast. The bedtools intersect tool can be used to filter out smORFs that are overlapping annotated coding regions. NCBI-blast can be used to filter out smORFs based on amino acid sequence similarity to annotated proteins.

## Expected outcomes

A successful Ribo-seq experiment should yield a library that is ∼155 bp in length. There should be a single peak without any broad shoulders on the bioanalyzer trace (Figure 5). However, like other sequencing library preparations adapter dimers can form, resulting in a PCR product with no RPF included. This adapter dimer will appear as a smaller peak of ∼120 bp and can bind to the flow cell and undergo sequencing, generating unusable data (troubleshooting 5). Prior to submitting samples for sequencing, library concentrations should be measured using the Qubit high sensitivity dsDNA assay kit. Yields after PCR amplification can typically range anywhere from 2 ng to ∼30 ng.

While a properly prepared Ribo-seq library peaks on a bioanalyzer at around 155 bp, this is not always indicative of successful enrichment of RPFs. Poor depletion of rRNAs can result in a low percentage of usable reads. Typically, the expected percentage of usable reads from the total amount of sequenced reads is around 20%, but this number may vary depending on the cell line or tissue type used.

During the processing of sequencing data, the generation of QC plots reveal the quality of Ribosome profiling data. The RPF read length distribution for high-quality data should show a peak at 28–29 nt (Figure 6).

In addition to an optimal read length distribution, the combined metagene plot of A-site offset corrected RPF reads should show 3-nt periodicity with enriched peaks at the translation initiation and termination sites (Figure 7), which indicate translating ribosomes within a CDS.

## Limitations

Ribo-seq sensitively detects the translation of smORFs. However, translated smORF detection by Ribo-seq does not report on the stability of translated proteins and therefore cannot guarantee that the smORF encoded microproteins are stable, functional biomolecules that can regulate biology. In addition, the detection of lowly translated smORFs can be noisy and it is recommended to collect biological replicates of Ribo-seq datasets.^8^

Lastly, the integrated analysis pipeline has some limitations compared to other more recently developed tools that predict smORF translation. RibORF v0.1 relies on candidate ORFs to be predefined to predict active translation, while more recent versions of RibORF and other newer Ribo-seq analysis tools have the ability to parse transcript databases for open reading frames and identify predicted translated ORFs *de novo* using Ribo-seq data.

## Troubleshooting

### Problem 1

The total RNA amount or concentration in lysate is low after lysis (step 10). There should ideally be at least 40 μg RNA in 200 μL of lysis buffer to proceed with RNase I digest to have sufficient starting material for library preparation.

### Potential solution

Depending on the cell line or tissue type, the amount of RNA harvested after lysis may not be sufficient for digest with RNase I. Increase the number of cells or amount of tissue used per sample to ensure that there is a sufficient RNA.

### Problem 2

The RPF read length distribution peaks at >29 nt and the percentage of in-frame reads by metagene analysis is not high (step 149). Longer RPF lengths correlate with insufficient RNase I digestion, thus leaving overhanging nucleotides on the ribosome footprint.

### Potential solution

When starting with a new cell or tissue type, it is beneficial to test multiple concentrations of RNase I during the digest step to find the optimal conditions. We recommend 0.375 U/μg as a useful starting point to test for most mammalian cell types.

### Problem 3

Denaturing TBE urea polyacrylamide gels used for RPF size selection and cDNA extraction show significant band “smiling,” making difficult to correctly cut out the correct band sizes (steps 78 and 118).

### Potential solution

TBE urea gels expire more quickly than non-denaturing gels. When used past the expiration date, these gels will have more band smiling as they get older. We recommend not using gels past the expiration date, but if users need to use expired gels then avoid using the edge wells as much as possible. For example, if you only need to run 8 lanes as in Figure 2, load lanes 2–9.

### Problem 4

The percentage of usable sequencing reads after filtering out reads that are too small and contaminant rRNA and tRNA reads is low (step 145). Ideally, the amount of sequenced RPF reads should make up at least 20% of the total number of reads sequenced.

### Potential solution

A low percentage of usable reads indicate poor enrichment of RPFs that is usually due to suboptimal rRNA depletion. While we recommend a starting probe concentration of 1.2× relative to digested RNA for most cell lines or sample types, the amount of probe used may need to be optimized. A probe concentration range of 1.2–1.5× has provided reasonable depletions in our experience using the riboPOOLs kit.

### Problem 5

The bioanalyzer QC profile has a peak of around 120 bp which are adapter dimers that need to be removed (step 112).

### Potential solution

Adapter dimers can appear in an amplified Ribo-seq library depending on a variety of factors including the yield of RPFs after the first gel extraction step and the concentration of the 3′ adapter used. However, when adapter dimers appear on the BioAnalyzer trace, they can be purified from the sample by gel extraction as in step 120.

## Resource availability

### Lead contact

Further information and requests for resources and reagents should be directed to and will be fulfilled by the lead contact, Thomas Martinez (t.martinez@uci.edu).

### Materials availability

This study did not generate new unique reagents.

## Data Availability

The raw datasets to repeat the Ribo-seq analysis are available at NCBI GEO accession number: GSE198109.
